# Identification of Alzheimer's Disease Progression Stages Using Topological Measures of Resting-State Functional Connectivity Networks: A Comparative Study

**DOI:** 10.1155/2022/9958525

**Published:** 2022-07-04

**Authors:** Zhanxiong Wu, Jinhui Wu, Xumin Chen, Xun Li, Jian Shen, Hui Hong

**Affiliations:** ^1^School of Electronic Information, Hangzhou Dianzi University, Hangzhou, Zhejiang 310018, China; ^2^School of Computer Science and Technology, Hangzhou Dianzi University, Hangzhou, Zhejiang 310018, China; ^3^Neurosurgery Department, The First Affiliated Hospital of Zhejiang University School of Medicine, Zhejiang University, Hangzhou, Zhejiang 310003, China

## Abstract

Resting-state functional magnetic resonance imaging (rs-fMRI) has been widely employed to examine brain functional connectivity (FC) alterations in various neurological disorders. At present, various computational methods have been proposed to estimate connectivity strength between different brain regions, as the edge weight of FC networks. However, little is known about which model is more sensitive to Alzheimer's disease (AD) progression. This study comparatively characterized topological properties of rs-FC networks constructed with Pearson correlation (PC), dynamic time warping (DTW), and group information guided independent component analysis (GIG-ICA), aimed at investigating the sensitivity and effectivity of these methods in differentiating AD stages. A total of 54 subjects from Alzheimer's Disease Neuroimaging Initiative (ANDI) database, divided into healthy control (HC), mild cognition impairment (MCI), and AD groups, were included in this study. Network-level (global efficiency and characteristic path length) and nodal (clustering coefficient) metrics were used to capture groupwise difference across HC, MCI, and AD groups. The results showed that almost no significant differences were found according to global efficiency and characteristic path length. However, in terms of clustering coefficient, 52 brain parcels sensitive to AD progression were identified in rs-FC networks built with GIG-ICA, much more than PC (6 parcels) and DTW (3 parcels). This indicates that GIG-ICA is more sensitive to AD progression than PC and DTW. The findings also confirmed that the AD-linked FC alterations mostly appeared in temporal, cingulate, and angular areas, which might contribute to clinical diagnosis of AD. Overall, this study provides insights into the topological properties of rs-FC networks over AD progression, suggesting that FC strength estimation of FC networks cannot be neglected in AD-related graph analysis.

## 1. Introduction

Alzheimer's disease (AD) is the most common cause for dementia, which has an insidious preclinical stage in which pathological tau accumulates slowly until clinical symptoms are observable in prodromal stages [1, 2]. For this reason, the neuroscience community is focusing on investigating early signs of AD which could lead to the development of validated biomarkers. Now, abundant functional magnetic resonance imaging (fMRI) studies have reported that cognitive impairments in AD were associated with abnormal functional connectivity (FC) interactions between different brain regions [3–5]. As spontaneous neuronal activity plays an important role in reflecting the brain's intrinsic mental state and human behavior [6], the investigation of brain resting-state FC (rs-FC) might facilitate our understanding of the neurophysiological mechanisms underlying AD progression. Therefore, resting-state fMRI (rs-fMRI) has been widely employed to study the alterations in neuronal activity of AD patients, with the measurement of resting brain synchronized activity through low frequency oscillations in the blood oxygen level-dependent (BOLD) signals [7, 8].

Currently, many rs-fMRI studies have showed altered functional connectivity in AD [9–11]. These studies assumed that FC patterns in the brain are static over the course of rs-fMRI scans. However, there is a growing consensus that the spontaneous fluctuations and correlations of signals between two distinct brain regions change over time, even in a resting state [12–15]. It is expected that dynamic properties of time-varying functional networks may offer additional information for understanding AD mechanisms. Conventional methods do not account for temporal variability and are not sensitive to associated connectivity changes in AD. As the brain works as a dynamically integrated network [16, 17], topologic characterization of dynamic FC networks may be better for revealing AD-linked FC alterations, which might be difficult to achieve in the traditional static network analysis of rs-fMRI [18].

At present, dynamic FC patterns have been primarily investigated using sliding-window technique [19–21], independent component analysis (ICA) [12, 22, 23], and leading eigenvector dynamics analysis (LEiDA) [24]. The sliding window technique has been repeatedly applied to explore how dynamic FC is affected by neurological disorders. In each windowed segment, between-region statistical association was usually estimated with Pearson correlation [11, 25] and dynamic time warping (DTW) [26, 27]. Pearson correlation directly measures the statistical linear relationships between pairs of BOLD series. DTW is an elastic matching algorithm, and as such can account for lag and shape differences between BOLD series [27]. ICA enables us to obtain dominant components by eliminating high-order statistical correlation of concatenated FC matrix [21]. Specially, group information guided ICA (GIG-ICA) showed promising potential for detecting altered brain dynamic functional connectivity [28, 29]. Differently, LEiDA captures the main orientation of BOLD signal phases over time by calculating the leading eigenvector for dynamic phase-locking matrix, which estimates the phase alignment between each pair of brain regions. These pioneering studies motivated us to comparatively investigate the sensitivity and effectivity of static and dynamic FC estimation methods in detecting alterations in rs-FC networks over AD progressive stages. In this study, after rs-FC networks were constructed using Pearson correlation, DTW, and GIG-ICA, topological analyses were performed. 54 subjects obtained from Alzheimer's Disease Neuroimaging Initiative (ANDI) database were included, consisting of 18 healthy controls (HCs), 18 mild cognition impairments (MCIs), and 18 ADs. The feasibility and effectivity of Pearson correlation, DTW, and GIG-ICA in exploring AD-related alterations were compared and discussed.

## 2. Materials and Methods

### 2.1. Data

Data used in our study were obtained from the Alzheimer's Disease Neuroimaging Initiative (ADNI) database (http://www.adni.loni.usc.edu). Detailed descriptions of the demographic and clinical features of the participants are provided in Table 1. The subjects were examined on a 3T MRI scanner (Magnetom Trio Tim, Siemens, Erlangen, Germany). MRI session included a high-resolution structural T1-weighted image (sagittal MPRAGE; TR = 2300 ms, TE = 2.98 ms, matrix size = 256 × 256 × 240, and isometric voxel 1 × 1 × 1 mm^3^) and a 10-min resting-state fMRI (TR = 3000 ms, TE = 16 ms, 128 × 128 × 40 matrix, and voxel size = 1.72 × 1.72 × 3 mm^3^). Participants were instructed to rest with eyes opened and stay awake in rs-fMRI scanning.

**Figure 1:**
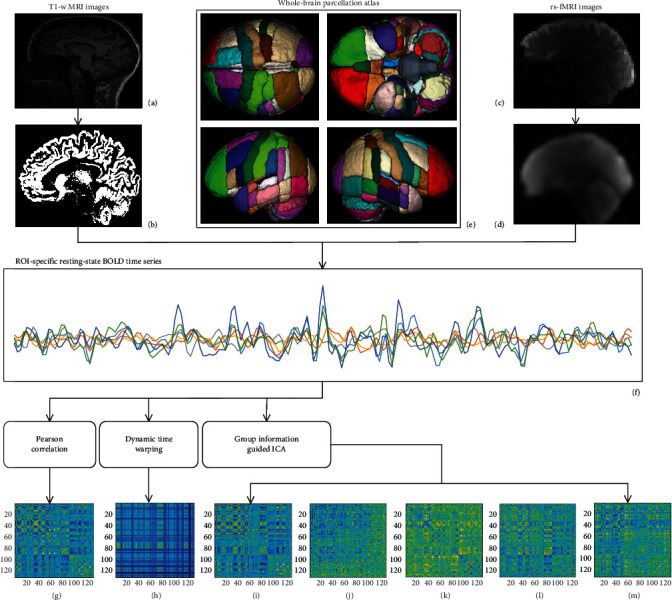
Flowchart for topological analyses of rs-FC networks. (a) T1-weighted MRI images were used to extract the tissues of gray matter, white matter, and cerebrospinal fluid. (b) Gray matter was coregistered into MNI space. (c) Original fMRI images were smoothed and coregistered into (d) MNI space. (e) Whole-brain parcellation atlas in MNI space was used to parcellate whole-brain into 132 parcels (Table 2), including cerebrum and cerebellum. (f) ROI-specific time serial was obtained by averaging BOLD signal timeseries within ROI voxels. (g) FC adjacent matrix computed with Pearson correlation. (h) FC adjacent matrix estimated with DTW. (i–m) Five ICA components extracted with GIG-ICA.

The entire processing flowchart in this study is shown in Figure 1. The tissues of gray matter, white matter, and cerebrospinal fluid were first segmented from T1-weighted MRI images. Gray matter was then coregistered into Montreal Neurosciences Institute (MNI) space. After original fMRI images were smoothed and coregistered into MNI space, whole-brain parcellation atlas in MNI space was employed to parcellate whole-brain into 132 parcels (Table 2), including cerebrum and cerebellum. ROI-specific time serial was obtained by averaging BOLD signal timeseries corresponding to the voxels within ROIs. At last, FC adjacent matrix computed with Pearson correlation, DTW, and GIG-ICA, respectively.

### 2.2. rs-FC Networks

In this work, CONN (functional connectivity toolbox, https://web.conn-toolbox.org/) and SPM12 (Statistical Parametric Mapping, https://www.fil.ion.ucl.ac.uk/spm/) were used to perform T1-weighted MRI and rs-fMRI preprocessing, including spatial coregistration, functional realignment and unwarp, slice-timing correction, outlier identification, segmentation and normalization, and smoothing [30, 31]. T1-weighted images were normalized into standard MNI space and segmented into gray matter, white matter, and CSF tissue classes using SPM12 unified segmentation and normalization procedure. rs-fMRI datasets were realigned using SPM12 realign and unwarp procedure, where all scans were coregistered and resampled to a reference image (the first scan) using b-spline interpolation. This procedure successfully addressed potential susceptibility distortion-by-motion interactions by estimating the derivatives of the deformation field with respect to head movement and resampling the functional data to match the deformation field of the reference image. Temporal misalignment between different slices of the functional data, induced by the sequential nature of fMRI acquisition protocol, was corrected using SPM12 slice-timing correction procedure, where the functional data was time-shifted and resampled using Sinc interpolation to match the time in the middle of each acquisition time. Last, functional data was smoothed using spatial convolution with a Gaussian kernel of 8 mm full width half maximum, in order to increase BOLD signal-to-noise ratio (SNR) and reduce the influence of residual variability in functional and gyral anatomy across subjects. Then, functional data was coregistered to the structural data using SPM12 intermodality coregistration procedure with a normalized mutual information cost function. This procedure estimated an optimal affine transformation between the reference functional image (mean BOLD signal) and the reference structural image (T1-weighted volume) that maximizes the mutual information between the two-modal imaging.

MNI-registered gray matter was divided into 132 regions (including 91 cortical regions, 15 subcortical regions, and 26 cerebellar regions) based on FSL Harvard-Oxford Atlas maximum likelihood cortical atlas and the automated anatomical labeling (AAL) template (Table 2) [32]. Then, whole-brain rs-FC networks were built, with gray matter parcels defined as nodes and the FC strength between the nodes considered as edge weights. Thus, weighted matrices were obtained for further analyses. In this study, for comparison, the edges of rs-FC network were estimated using three methods, including Pearson correlation, DTW, and GIG-ICA (https://www.nitrc.org/projects/gig-ica/). Static FC strength was estimated over entire rs-fMRI scan in terms of Pearson correlation coefficient and DTW similarity [27]. After FC matrix was obtained from covariance matrix with a window width of 20 TRs [33], the first (dominant) component extracted by GIG-ICA from the concatenated dynamic FC matrices would be used for topological analysis. As group information captured by standard ICA on the group level is used as guidance to compute individual subject specific independent components (ICs), GIG-ICA is able to obtain subject-specific ICs with better spatial correspondence, higher spatial, and temporal accuracy [28, 34].

### 2.3. Topological Metrics of rs-FC Networks

For topological analysis, we focused on global efficiency, characteristic path length, and clustering coefficient, which were calculated based on the estimated FC adjacency matrices using BCT toolbox [35]. Our goal is to use these metrics to investigate the FC disruption caused by AD progression. The metrics are summarized as follows.

Global efficiency is inversely proportional to the average shortest path length. This metric measures how globally efficient a network is in the sense of connecting distant nodes together [35, 36].

(1) $$\begin{array}{c} E=\frac{1}{N}\underset{i}{\sum }\frac{1}{N-1}\frac{1}{\sum_{j\neq i}d_{ij}}, \end{array}$$

where *d*_*ij*_ is the shortest path length between node *i* and node *j*. *N* is the number of nodes in a network.

Characteristic path length indicates a lower capacity to integrate information using shortest path routing, revealing the integration of a network structure [35].

(2) $$\begin{array}{c} L=\frac{1}{N}\underset{i}{\sum }\frac{\sum_{i\neq j}d_{ij}}{N-1}. \end{array}$$

The clustering coefficient indicates the extent of local interconnectivity or cliquishness in a network. It is defined as the ratio of the number of triangles a given node belongs to over the total number of triangles it could belong to [37].

(3) $$\begin{array}{c} C=\frac{1}{N}\underset{i}{\sum }\frac{2t_{i}}{k_{i}\left(k_{i}-1\right)}, \end{array}$$

where *t*_*i*_ is the number of triangles around node and *k*_*i*_ is the node degree.

### 2.4. Statistical Analysis

Once the graph measures were extracted, we statistically evaluated groupwise difference. After whole-brain static and dynamic FC networks of each subject was built, groupwise differences of topologic metrics were assessed using one-way analysis of covariance (ANCOVA) tests with age and gender as covariates [22, 38]. As an extension of analysis of variance (ANOVA), ANCOVA provides a way of statistically controlling the effect of covariables. In this study, *p* value less than 0.05 (uncorrected) was considered statistically significant.

## 3. Results

### 3.1. rs-FC Adjacent Matrices

**Figure 2:**
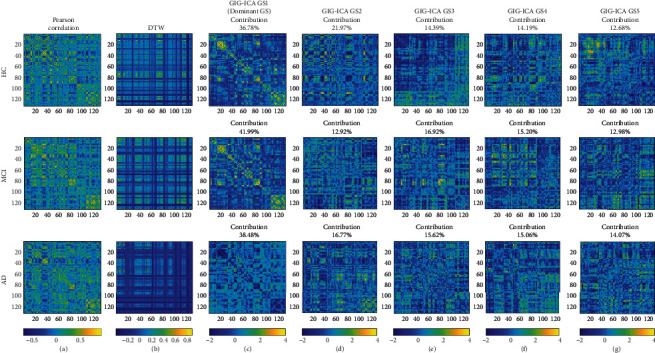
rs-FC adjacent matrices. (a) FC matrices computed with Pearson correlation, and (b) was obtained with DTW. (c) Dominant component of GIG-ICA decomposition. From top to bottom, the first, second, and third rows denote examples from subjects in HC, MCI, and AD groups, respectively.

Figure 2 shows examples of whole-brain FC estimation from HC, MCI, and AD subjects. In this work, rs-FC adjacent matrices were estimated using Pearson correlation, DTW, and GIG-ICA. Figures 2(d)–2(g) denote group-level FC states (GSs), and Figure 2(c) is the dominant GSs which contribute the most information across the entire time-varying connectivity patterns. Figure 2(a) was computed with Pearson correlation, and Figure 2(b) was obtained with DTW.

### 3.2. Topological Measures

**Figure 3:**
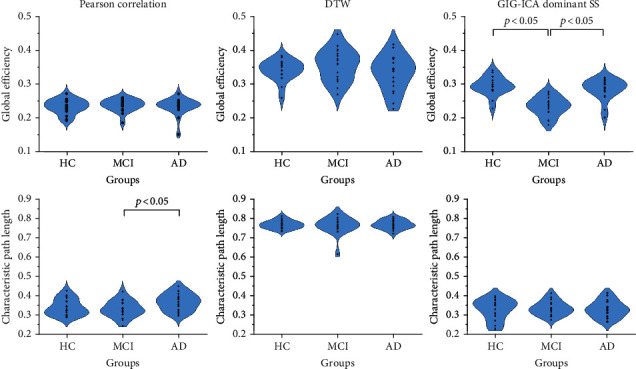
Violin plots of ANCOVA analyses on network-level topological metrics, including global efficiency and characteristic path length. No significant differences were found in rs-FC networks constructed with DTW, in terms of the both metrics. As for Pearson correlation, only MCI and AD groups were significantly identified according to the metric of characteristic path length. In FC networks constructed with GIG-ICA, significant differences between HC, AD, and MCI groups were found in terms of global efficiency.

As shown in Figure 3, no significant differences were found in FC networks constructed with DTW, in terms of the both network-level metrics. In FC networks built with Pearson correlation, only MCI and AD groups were significantly identified according to the metric of characteristic path length. In GIG-ICA dominant component networks, significant differences between HC, AD, and MCI groups were found in terms of global efficiency. Overall, significant groupwise differences could hardly be detected in terms of characteristic path length. The mean and standard deviation values of the network-level topological metrics are reported in Table 3.

**Figure 4:**
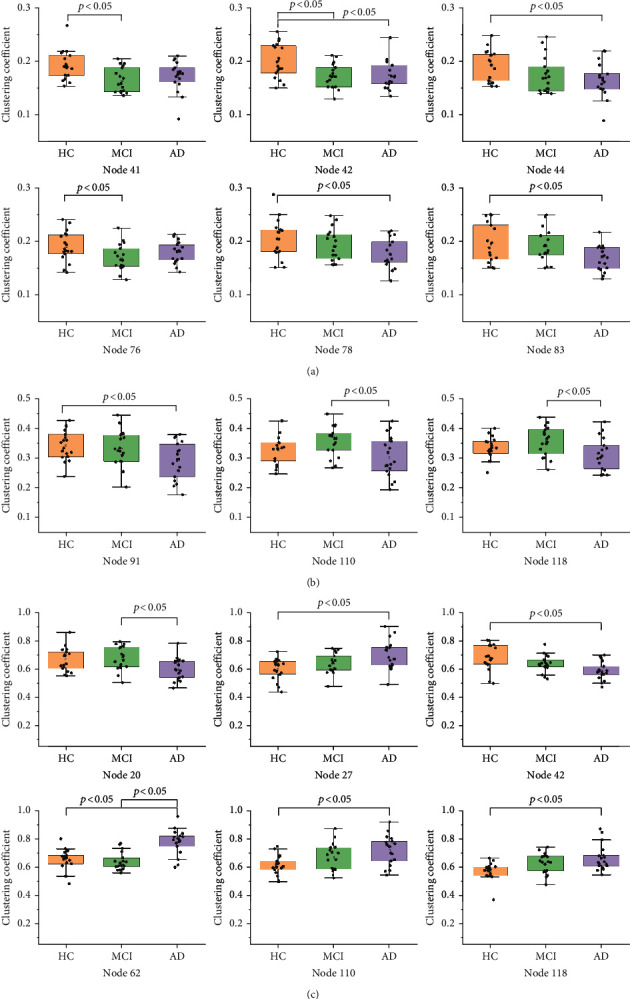
Box plots of ANCOVA analyses on clustering coefficient. Significant differences across HC, MCI, and AD stages were found in 6 brain parcels of rs-FC networks constructed with (a) Pearson correlation, in 3 brain parcels of rs-FC networks constructed with (b) DTW, and in 52 brain parcels of (c) GIG-ICA dominant component networks. For simplification, only 6 nodes were displayed in (c).

**Figure 5:**
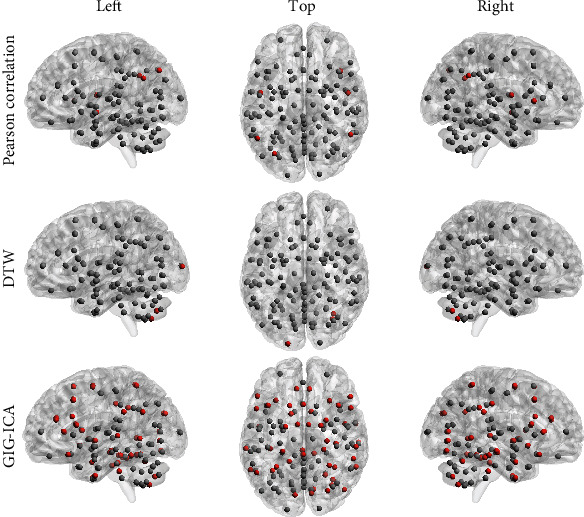
Distribution of the brain parcels (red dots) that exhibit significant groupwise differences over HC, MCI, and AD stages, in terms of nodal clustering coefficient. For the node indexes and names, refer to Tables 2 and 7.

Figure 4 shows ANCOVA results regarding the nodal metric of clustering coefficient. The box plots of ANCOVA are depicted in Figure 4, and the metric values (mean ± standard) are reported in Tables 4–6. In the rs-FC networks built using Pearson correlation, significant differences across HC, MCI, and AD groups were found for 6 brain parcels, including right angular gyrus (41), left angular gyrus (42), left superior division of lateral occipital cortex (44), right frontal operculum cortex (76), right central opercular cortex (78), and left planum polare (83) (Tables 2 and 7). Only three brain parcels were significantly identified in DTW-constructed networks, namely, left occipital pole (91), right cerebellum crus2 (110), and right cerebellum 7 (118) (Tables 2 and 7). In GIG-ICA dominant component networks, we found that total 52 brain parcels (Tables 2 and 7) exhibit significantly different clustering coefficient across HC, MCI, and AD groups.  Figure 5 shows the 3D distribution of these significantly identified brain parcels (red dots).

## 4. Discussion

At present, there are a few computational models to estimate FC strength between pairs of brain parcels, which were used to investigate FC alterations in various neurological disorders. In this study, we performed topological analyses on rs-FC networks of 18 HCs, 18 MCIs, and 18 Ads, aiming to finding a FC strength computational method sensitive to AD progression. Groupwise differences in global and nodal metrics were examined using ANCOVA tests with gender and age as covariates. Our results showed that almost no significant differences were observed across HC, MCI, and AD groups in terms of global metrics, and that much more affected brain regions by AD were revealed according clustering coefficient extracted from GIG-ICA dominant rs-FC networks. These findings provide new insights into understanding of the macroscopic network mechanisms underlying AD progression.

The human brain is a complex and interconnected network characterized by an efficient small-world network with high local clustering [39, 40]. Compared with healthy controls, MCI and AD patients exhibit a disruption of the global integration of brain networks [5]. However, in this study, almost no significant differences (*p* < 0.05, uncorrected) among HC, MCI, and AD groups were observed in terms of network-level metrics derived from rs-FC networks, including global efficiency and characteristic path length (Figure 3 and Table 3). In other words, global topologic metrics extracted from rs-FC networks are not sensitive to AD progression. Interestingly, in GIG-ICA dominant rs-FC networks, global efficiency of MCI group significantly decreased compared with HC and AD groups (*p* < 0.05, uncorrected). By comparison, significant intergroup differences have been identified in structural connectivity (SC) networks [3, 41, 42]. This may due to different nature of FC and SC brain networks [43, 44]. FC reflects neuronal synchronization between brain regions and is vulnerable to physiologic and psychologic conditions. SC serves as the substrate for FC, with relative stability.

As a measure of local interconnectivity, clustering coefficient was estimated to examine FC disruption caused by AD. For rs-FC networks constructed with Pearson correlation and DTW, significant difference across HC, MCI, and AD groups were observed only in 6 (Table 4) and 3 (Table 5) regions. However, statistically significant differences among HC, MCI, and AD groups were observed in total 52 nodes of GIG-ICA dominant rs-FC networks (Table 6), mainly distributed in temporal, cingulate, and angular areas. [45] reported that AD patients exhibit the posterior medial temporal neurodegeneration that was associated with episodic memory disturbance. In line with the previous finding, our results also showed AD patients have low clustering coefficient in the temporal gyrus and cingulate region [46–48]. However, some brain parcels displayed higher clustering coefficient over AD progression (Figure 4) such as right anterior division of inferior temporal gyrus (27), right anterior division of parahippocampal gyrus (62), right cerebellum crus2 (110), and right cerebellum 7 (118). This phenomenon can be interpreted as a compensatory mechanism to maintain normal cognitive function under AD pathology [12, 49, 50], which deserves further study. From the ANCOVA results, compared with Pearson correlation and DTW, we could conclude that GIG-ICA was more sensitive to AD progression. This may be attributed to that dynamic rs-FC and the derived FC topological metrics might help reveal changes in macroscopic neural interaction patterns underlying AD progression [23]. Pearson correlation and DTW are here applied to measure static FC. Dynamic FC reveals temporal patterns of FC and provides additional information to static FC, confirming that it might be an efficient way to facilitate our understanding of the underlying neurophysiological mechanisms of AD.

Several limitations should be addressed in the future work. First, an empirically validated fixed sliding window of 20 TRs was selected in GIG-ICA. The selection of sliding window size is still inconclusive and may impact the evaluation of rs-FC connectivity strength. FC changes with separate windows of various window lengths will be evaluated in the future study. Second, the sample sizes included in the current study are relatively small, and the replication study with more participants needs to be performed in the future to verify our findings. Third, the whole brain was divided coarsely into 132 regions based on FSL Harvard-Oxford atlas and AAL template for functional brain network construction. Different parcellation strategies are required to validate our findings in future studies. Finally, the association between the disrupted brain functional networks and the neurological conditions was not explored. Future longitudinal studies are necessary to explore potential altered dynamic FC as a clinical biomarker.

## 5. Conclusion

rs-FC has been often used to identify abnormal brain connectivity patterns in AD cohorts and to understand mechanisms of abnormal brain function, attempting to comprehensively explore the potential utility of rs-FC as a biomarker for AD progression. In summary, this study revealed that more affected regions can be significantly identified in terms of nodal clustering coefficient extracted from GIG-ICA dominant networks. As GIG-ICA dominant component was extracted from the concatenated time-varying rs-FC matrices, it may be concluded that dynamic FC analysis has the potential to provide more reliable and significant scientific findings than static FC studies. Our results also indicated the potential role of clustering coefficient in determination of early-stage AD patients, providing a clue to AD diagnosis and monitoring in clinical applications. In the next step, joint analysis between structural and functional networks could be a better way to reveal brain connectivity alterations over AD progression.

## Figures and Tables

**Table 1:** Demographic information for HC, MCI, and AD subjects.

	Number	Age (mean ± standard)	Gender	MMSE score (mean ± standard)	CDR global score (mean ± standard)
HC	18	69.11 ± 7.87	15F, 3M	29.1 ± 1.0	0.0 ± 0.0
MCI	18	72.00 ± 5.89	12F, 6M	27.0 ± 1.8	0.5 ± 0.0
AD	18	73.17 ± 7.85	6F, 12M	23.3 ± 2.1	0.7 ± 0.3

**Table 2:** Index and name of 132 brain parcels, including 91 cortical, 15 subcortical, and 26 cerebellar subregions. This parcellation atlas was based on FSL Harvard-Oxford and the automated anatomical labeling (AAL) template.

Index/name	Index/name	Index/name	Index/name	Index/name	Index/name
1 Frontal pole right	23 Middle temporal gyrus, posterior division right	45 Lateral occipital cortex, inferior division right	67 Lingual gyrus left	89 Supracalcarine cortex left	111 Cerebellum 3 left
2 Frontal pole left	24 Middle temporal gyrus, posterior division left	46 Lateral occipital cortex, inferior division left	68 Temporal fusiform cortex, anterior division right	90 Occipital pole right	112 Cerebellum 3 right
3 Insular cortex right	25 Middle temporal gyrus, temporooccipital part right	47 Intracalcarine cortex right	69 Temporal fusiform cortex, anterior division left	91 Occipital pole left	113 Cerebellum 4, 5 left
4 Insular cortex left	26 Middle temporal gyrus, temporooccipital part left	48 Intracalcarine cortex left	70 Temporal fusiform cortex, posterior division right	92 Thalamus right	114 Cerebellum 4, 5 right
5 Superior frontal gyrus right	27 Inferior temporal gyrus, anterior division right	49 Frontal medial cortex	71 Temporal fusiform cortex, posterior division left	93 Thalamus left	115 Cerebellum 6 left
6 Superior frontal gyrus left	28 Inferior temporal gyrus, anterior division left	50 Supplementary motor cortex right	72 Temporal occipital fusiform cortex right	94 Caudate right	116 Cerebellum 6 right
7 Middle frontal gyrus right	29 Inferior temporal gyrus, posterior division right	51 Supplementary motor cortex left	73 Temporal occipital fusiform cortex left	95 Caudate left	117 Cerebellum 7 left
8 Middle frontal gyrus left	30 Inferior temporal gyrus, posterior division left	52 Subcallosal cortex	74 Occipital fusiform gyrus right	96 Putamen right	118 Cerebellum 7 right
9 Inferior frontal gyrus, pars triangularis right	31 Inferior temporal gyrus, temporooccipital part right	53 Paracingulate gyrus right	75 Occipital fusiform gyrus left	97 Putamen left	119 Cerebellum 8 left
10 Inferior frontal gyrus, pars triangularis left	32 Inferior temporal gyrus, temporooccipital part left	54 Paracingulate gyrus left	76 Frontal operculum cortex right	98 Pallidum right	120 Cerebellum 8 right
11 Inferior frontal gyrus, pars opercularis right	33 Postcentral gyrus right	55 Cingulate gyrus, anterior division	77 Frontal operculum cortex left	99 Pallidum left	121 Cerebellum 9 left
12 Inferior frontal gyrus, pars opercularis left	34 Postcentral gyrus left	56 Cingulate gyrus, posterior division	78 Central opercular cortex right	100 Hippocampus right	122 Cerebellum 9 right
13 Precentral gyrus right	35 Superior parietal lobule right	57 Precuneous cortex	79 Central opercular cortex left	101 Hippocampus left	123 Cerebellum 10 left
14 Precentral gyrus left	36 Superior parietal lobule left	58 Cuneal cortex right	80 Parietal operculum cortex right	102 Amygdala right	124 Cerebellum 10 right
15 Temporal pole right	37 Supramarginal gyrus, anterior division right	59 Cuneal cortex left	81 Parietal operculum cortex left	103 Amygdala left	125 Vermis 1, 2
16 Temporal pole left	38 Supramarginal gyrus, anterior division left	60 Frontal orbital cortex right	82 Planum polare right	104 Accumbens right	126 Vermis 3
17 Superior temporal gyrus, anterior division right	39 Supramarginal gyrus, posterior division right	61 Frontal orbital cortex left	83 Planum polare left	105 Accumbens left	127 Vermis 4, 5
18 Superior temporal gyrus, anterior division left	40 Supramarginal gyrus, posterior division left	62 Parahippocampal gyrus, anterior division right	84 Heschl's gyrus right	106 Brain-stem	128 Vermis 6
19 Superior temporal gyrus, posterior division right	41 Angular gyrus right	63 Parahippocampal gyrus, anterior division left	85 Heschl's gyrus left	107 Cerebellum crus1 left	129 Vermis 7
20 Superior temporal gyrus, posterior division left	42 Angular gyrus left	64 Parahippocampal gyrus, posterior division right	86 Planum temporale right	108 Cerebellum crus1 right	133 Vermis 8
21 Middle temporal gyrus, anterior division right	43 Lateral occipital cortex, superior division right	65 Parahippocampal gyrus, posterior division left	87 Planum temporale left	109 Cerebellum crus2 left	131 Vermis 9
22 Middle temporal gyrus, anterior division left	44 Lateral occipital cortex, superior division left	66 Lingual gyrus right	88 Supracalcarine cortex right	110 Cerebellum crus2 right	132 Vermis 10

**Table 3:** Network-level topological metrics (mean ± standard) derived from rs-FC networks, including global efficiency and characteristic path length.

	Pearson correlation			DTW			GIG-ICA dominant SS		
	HC	MCI	AD	HC	MCI	AD	HC	MCI	AD
Global efficiency	0.23 ± 0.02	0.24 ± 0.02	0.23 ± 0.03	0.34 ± 0.03	0.35 ± 0.05	0.33 ± 0.05	0.29 ± 0.03	0.23 ± 0.03	0.28 ± 0.03
Characteristic path length	0.34 ± 0.04	0.33 ± 0.04	0.37 ± 0.04	0.77 ± 0.02	0.76 ± 0.05	0.77 ± 0.02	0.33 ± 0.05	0.34 ± 0.04	0.33 ± 0.04

**Table 4:** Clustering coefficient (mean ± standard) of the nodes that can significantly differentiate HC, MCI, and AD groups. The edge weights were estimated with Pearson correlation.

	N41	N42	N44	N76	N78	N83
HC	0.19 ± 0.03	0.20 ± 0.03	0.19 ± 0.03	0.19 ± 0.03	0.21 ± 0.04	0.20 ± 0.04
MCI	0.16 ± 0.02	0.17 ± 0.02	0.17 ± 0.03	0.17 ± 0.02	0.19 ± 0.03	0.19 ± 0.03
AD	0.17 ± 0.03	0.18 ± 0.03	0.16 ± 0.03	0.18 ± 0.02	0.18 ± 0.03	0.17 ± 0.02

**Table 5:** Clustering coefficient (mean ± standard) of the nodes that can significantly differentiate HC, MCI, and AD groups. The edge weights were estimated with DTW.

	N91	N110	N118
HC	0.34 ± 0.05	0.33 ± 0.05	0.33 ± 0.04
MCI	0.33 ± 0.06	0.35 ± 0.05	0.36 ± 0.05
AD	0.29 ± 0.06	0.30 ± 0.07	0.31 ± 0.05

**Table 6:** Clustering coefficient (mean ± standard) of the nodes that can significantly differentiate HC, MCI, and AD groups. The edge weights were computed with GIG-ICA dominant component.

	N6	N7	N8	N9	N10	N11	N12	N20	N21	N23	N24	N25	N26
HC	0.67 ± 0.08	0.70 ± 0.10	0.67 ± 0.07	0.70 ± 0.07	0.69 ± 0.08	0.70 ± 0.09	0.70 ± 0.08	0.67 ± 0.08	0.67 ± 0.08	0.68 ± 0.10	0.69 ± 0.06	0.70 ± 0.10	0.68 ± 0.08
MCI	0.58 ± 0.07	0.60 ± 0.07	0.59 ± 0.05	0.59 ± 0.06	0.60 ± 0.05	0.60 ± 0.07	0.60 ± 0.05	0.68 ± 0.08	0.62 ± 0.08	0.61 ± 0.07	0.62 ± 0.06	0.58 ± 0.05	0.61 ± 0.06
AD	0.58 ± 0.08	0.63 ± 0.12	0.64 ± 0.12	0.63 ± 0.07	0.64 ± 0.10	0.70 ± 0.10	0.67 ± 0.10	0.60 ± 0.08	0.71 ± 0.10	0.59 ± 0.07	0.59 ± 0.07	0.56 ± 0.06	0.58 ± 0.08
	N27	N35	N36	N37	N39	N42	N45	N47	N50	N51	N53	N54	N55
HC	0.60 ± 0.08	0.57 ± 0.05	0.58 ± 0.07	0.62 ± 0.09	0.70 ± 0.09	0.68 ± 0.09	0.63 ± 0.08	0.64 ± 0.08	0.65 ± 0.08	0.66 ± 0.08	0.70 ± 0.10	0.70 ± 0.08	0.65 ± 0.08
MCI	0.64 ± 0.07	0.60 ± 0.06	0.62 ± 0.07	0.61 ± 0.08	0.62 ± 0.07	0.64 ± 0.06	0.64 ± 0.09	0.64 ± 0.06	0.60 ± 0.06	0.56 ± 0.07	0.63 ± 0.08	0.62 ± 0.10	0.63 ± 0.07
AD	0.70 ± 0.10	0.70 ± 0.09	0.67 ± 0.08	0.72 ± 0.10	0.76 ± 0.09	0.59 ± 0.06	0.56 ± 0.07	0.57 ± 0.08	0.68 ± 0.12	0.64 ± 0.10	0.57 ± 0.09	0.55 ± 0.06	0.54 ± 0.09
	N56	N58	N59	N60	N61	N62	N64	N65	N70	N71	N72	N73	N74
HC	0.64 ± 0.07	0.61 ± 0.07	0.61 ± 0.08	0.69 ± 0.09	0.65 ± 0.07	0.65 ± 0.08	0.66 ± 0.04	0.64 ± 0.06	0.59 ± 0.05	0.58 ± 0.09	0.61 ± 0.07	0.60 ± 0.08	0.63 ± 0.09
MCI	0.52 ± 0.04	0.66 ± 0.10	0.66 ± 0.07	0.57 ± 0.08	0.57 ± 0.09	0.64 ± 0.06	0.58 ± 0.05	0.58 ± 0.05	0.61 ± 0.06	0.57 ± 0.08	0.64 ± 0.07	0.65 ± 0.07	0.67 ± 0.08
AD	0.60 ± 0.11	0.57 ± 0.06	0.57 ± 0.08	0.60 ± 0.09	0.59 ± 0.07	0.78 ± 0.09	0.71 ± 0.09	0.70 ± 0.09	0.66 ± 0.08	0.65 ± 0.07	0.56 ± 0.06	0.56 ± 0.05	0.56 ± 0.06
	N95	N96	N98	N99	N100	N104	N106	N110	N112	N113	N116	N118	N125
HC	0.65 ± 0.07	0.64 ± 0.10	0.60 ± 0.07	0.62 ± 0.07	0.59 ± 0.08	0.64 ± 0.07	0.64 ± 0.07	0.61 ± 0.07	0.64 ± 0.08	0.63 ± 0.09	0.60 ± 0.10	0.57 ± 0.06	0.61 ± 0.07
MCI	0.62 ± 0.08	0.54 ± 0.08	0.50 ± 0.07	0.53 ± 0.05	0.56 ± 0.08	0.57 ± 0.04	0.61 ± 0.07	0.67 ± 0.10	0.60 ± 0.05	0.63 ± 0.06	0.72 ± 0.10	0.63 ± 0.07	0.56 ± 0.07
AD	0.56 ± 0.05	0.58 ± 0.07	0.59 ± 0.06	0.59 ± 0.10	0.65 ± 0.09	0.60 ± 0.08	0.69 ± 0.09	0.72 ± 0.10	0.68 ± 0.09	0.71 ± 0.10	0.66 ± 0.09	0.66 ± 0.09	0.66 ± 0.06

**Table 7:** rs-FC network nodes which exhibit significant groupwise difference across HC, MCI, and AD groups.

	Node index
Pearson correlation	41, 42, 44, 76, 78, 83
DTW	91, 110, 118
GIG-ICA	6, 7, 8, 9, 10, 11, 12, 20, 21, 23, 24, 25, 26, 27, 35, 36, 37, 39, 42, 45, 47, 50, 51, 53, 54, 55, 56, 58, 59, 60, 61, 62, 64, 65, 70, 71, 72, 73, 74, 95, 96, 98, 99, 100, 104, 106, 110, 112, 113, 116, 118, 125

## Data Availability

The data used to support the findings of this study can be requested from the corresponding author.
